# Association of number of chronic physical multimorbidities and health-related quality of life in anxiety/stress-related disorders compared with schizophrenia-spectrum and mood/affective disorders

**DOI:** 10.1192/j.eurpsy.2026.11233

**Published:** 2026-07-31

**Authors:** K. Matić, Ž. Bajić, K. Bosak, S. Vuk-Pisk, V. Grošić, B. Ivana, I. Simunovic Filipcic, I. Filipcic

**Affiliations:** 1 University Psychiatric Clinic Sveti Ivan, Zagreb; 2 Faculty of Dental Medicine and Health, Josip Juraj Strossmayer University of Osijek, Osijek; 3 University of Applied Health Science; 4 Department of Psychiatry and Psychological Medicine, University Hospital Center Zagreb, Zagreb, Croatia

## Abstract

**Introduction:**

Chronic physical multimorbidity (CPM) impairs health-related quality of life (HRQoL) in psychiatric patients, but whether the rate of deterioration with increasing somatic burden differs by diagnosis is unclear. We focused on anxiety/stress-related disorders, using schizophrenia-spectrum (SSD) and mood/affective disorders as comparators.

**Objectives:**

To test whether the CPM–HRQoL association in anxiety/stress-related disorders differs from the association in SSD and mood/affective disorders.

**Methods:**

Cross-sectional study on patients treated in tertiary psychiatric institution in Croatia. CPM was modelled as a continuous count of conditions. Outcomes were SF-36 Physical (PCS) and Mental (MCS) Component Summary scores. Linear regression with robust standard errors included diagnosis × comorbidity interaction and covariates (gender, age, education, employment, smoking). BMI was not entered because obesity was part of the comorbidity count.

**Results:**

The sample included 313 patients with anxiety/stress-related disorders, 314 patients diagnosed with SSD and 230 with mood/affective disorders. In diagnosis-stratified unadjusted models, each additional chronic condition was associated with MCS change of −5.86 points in SSD (p < 0.001), −2.99 in mood/affective (p = 0.001), and −2.48 in anxiety/stress (p < 0.001). In the adjusted interaction model, the reference slope in SSD was −5.31 points per additional condition. Slope differences versus SSD were +2.32 for mood/affective (p = 0.116) and +2.66 for anxiety/stress (p = 0.044), indicating a significant MCS decline with increasing multimorbidity in anxiety/stress and an intermediate, non-significant decline in mood/affective. Model R^2^ = 0.16. In analogous continuous PCS models, slopes were consistently negative and did not differ significantly by diagnosis.

**Conclusions:**

CPM is associated with lower SF-36 MCS across psychiatric diagnoses. Compared with SSD, anxiety/stress-related disorders show a significantly larger MCS decline per additional condition, while mood/affective disorders are intermediate. For PCS, deterioration per condition appears similar across diagnoses. Findings support integrated somatic–psychiatric care and suggest that the mental-health impact per added physical condition depends on diagnostic context, particularly in anxiety/stress-related disorders.

**Disclosure of Interest:**

None Declared

